# Nutrition and Exercise in a Case of Carnitine Palmitoyl-Transferase II Deficiency

**DOI:** 10.3389/fphys.2021.637406

**Published:** 2021-03-17

**Authors:** Mauro Parimbelli, Elena Pezzotti, Massimo Negro, Luca Calanni, Silvia Allemano, Marco Bernardi, Angela Berardinelli, Giuseppe D’Antona

**Affiliations:** ^1^Criams-Sport Medicine Centre Voghera, University of Pavia, Pavia, Italy; ^2^Child Neuropsychiatry, IRCCS Mondino Foundation, Pavia, Italy; ^3^Department of Physiology and Pharmacology, University of Rome “La Sapienza”, Rome, Italy; ^4^Department of Public Health, Experimental and Forensic Medicine, University of Pavia, Pavia, Italy

**Keywords:** training, fatty acids metabolism, carnitine acetyl transferase, resistance exercise and interval training, nutrition

## Abstract

In the mild subtype of inherited carnitine palmitoyltransferase II (CPTII) deficiency, muscular mitochondrial fatty acid β-oxidation is impaired. In this condition, interventions involve daily dietary restriction of fats and increase of carbohydrates, whereas physical exercise is commonly contraindicated due to the risk of muscle pain and rhabdomyolysis. We present the case of a 14-year-old female with CPTII deficiency who underwent a 1-h session of unsupervised exercise training for 6 months, 3 days per week, including interval and resistance exercises, after diet assessment and correction. Before and after intervention, the resting metabolic rate (RMR) and respiratory quotient (RQ) were measured by indirect calorimetry, and a cardiopulmonary exercise test (CPET, 10 W/30 s to exhaustion) was performed. Interval training consisted of a 1 min run and a 5 min walk (for 15 min progressively increased to 30 min). During these efforts, the heart rate was maintained over 70% HR max corresponding to respiratory exchange ratio (RER) of 0.98. Resistance training included upper/lower split workouts (3 sets of 8 repetitions each, with 2 min rest between sets). Blood CK was checked before and 36 h after two training sessions chosen randomly without significant difference. After training, RMR increased (+8.1%) and RQ lowered into the physiological range (from 1.0 to 0.85). CPET highlighted an increase of peak power output (+16.7%), aerobic performance (VO_2_ peak, 8.3%) and anaerobic threshold (+5.7%), oxygen pulse (+4.5%) and a much longer isocapnic buffering duration (+335%). No muscle pain or rhabdomyolysis was reported. Results from our study highlight that training based on short-duration high-intensity exercise improves overall metabolism and aerobic fitness, thus being feasible, at least in a case of CPTII deficiency.

## Introduction

The carnitine palmitoyltransferase (CPT) system includes two enzymes, CPTI and CPTII, located in the outer and inner mitochondrial membranes, respectively, and involved in the transportation of long-chain fatty acids (LCFA) into the mitochondria (Lehmann et al., 2017).

CPTII deficiency is the most frequent muscular inherited disorder of the muscle fatty acid metabolism resulting in reduced LCFA oxidation as an energy source, both at rest and during muscular activities. CPTII deficiency leads to a spectrum of clinical conditions ranging from a lethal neonatal disorder, a severe infantile hepatocardiomuscular disorder and a mild myopathic form (Boemer et al., 2016). Most of the patients (around 90%) carry a p. S113L mutation in homozygous or heterozygous state with an allele frequency of 60–70% and more than 60 mutations (Isackson et al., 2006; Fanin et al., 2012). CPTII deficiency is considered to be an autosomal recessive disorder, so incidence is equal in males and females. Of importance, more than 75% of patients reported so far are males, and it is currently unknown whether the gender predominance is due to sex-related differences in exercise activities, hormonal factors, or both (Joshi and Zierz, 2020).

Clinical symptoms of the disease occur more frequently during infancy (1–12 years old) than in adolescence (13–22 years old) and adulthood (>22 years old) (Bonnefont et al., 2004). Muscle weakness, fatigue, myalgia, pain, and rhabdomyolysis often represent intermittent manifestations of the mild myopathic form and the exposure to cold, fasting, fever, and prolonged exercise may represent their triggering factors (Bonnefont et al., 2004).

The biochemical consequences of the disease-causing mutations are still debated. Previous studies highlighted that the enzyme activity ranges from undetectable (Bank et al., 1975; Layzer et al., 1980) up to normal in CPTII-deficient muscles (Trevisan et al., 1984). As a matter of fact, an impaired muscular utilization of LCFA has been found in CPTII patients, particularly during long-lasting low-intensity exercises, offset by increased glycogenolysis, whereas a normal fat utilization may be found at rest (Ørngreen et al., 2005).

In CPTII deficiency, to prevent symptoms and rhabdomyolysis, the current interventions involve a daily dietary restriction of fats and increased carbohydrate intake. Glucose infusion is adopted whenever the patient has a fever or infections that raise the metabolic demand. The suggested nutritional regimen results in a short-term increase of the exercise tolerance but recurrent rhabdomyolysis has been also observed in these cases (Deschauer et al., 2005). Therefore, restriction of physical activity is generally recommended (Roe et al., 2008).

Despite these premises, we speculate that a specific type of training, based on short duration contractions and resistance exercises, can be beneficial also for CPTII deficient patients. In fact, it is widely known that the energy sources used during exercise strictly depend on the duration and percentage of the maximal sustainable load (Berardinelli and D’Antona, 2019). In particular, integrated processes operate to satisfy the energy requirements of muscles during exercise, and they include the anaerobic alactic system—involved in splitting stored phosphagens, ATP, and phosphocreatine (PCr)—the anaerobic lactic system (involved in anaerobic breakdown of carbohydrate to lactic acid through glycolysis), and the aerobic system (involved in splitting of carbohydrates and fats in the presence of oxygen). Overall, the anaerobic pathways generate ATP at higher rates than the aerobic system. In fact, it has been demonstrated that degradation of PCr and glycolysis provide energy over the initial time frame of a brief intense exercise. In this condition, the rate of PCr degradation reaches its maximum in little more than 1 s whereas the energy production from glycolysis reaches it in around 5 s, being maintained at this level for several s (Maughan et al., 1997). When the intense activity is maintained for 2–3 min, alactic sources contribute for 20–30% of energy release, and the remaining need is mainly sustained by the activation of glycolysis (Medbø et al., 1988; Bangsbo et al., 1990; Saltin, 1990), whereas an equal contribution of the anaerobic and aerobic systems sustains efforts lasting more than 3 min (Fox et al., 1993) or between 2 and 3 min (Bangsbo et al., 1990). Based on these considerations, in order to force the skeletal muscle bioenergetics to predominantly use PCr and carbohydrates over fats as energy sources, the variables to be strictly considered are duration (short) and intensity (high) of the exercise (Baker et al., 2010). Therefore, considering that in short-duration, high-intensity exercise the high-energy phosphates and glycolytic systems are prominently used, we hypothesized that the application of such training could be useful in presence of CPTII deficiency, when a correct dietary regimen has been previously set up.

### Case Presentation and Clinical Evaluations

We report here the case of a 14-year-old girl complaining of lower-limb weakness and myalgia following physical exercise at school. The patient and her parents gave their written consent to participate in the study and for the data to be published after a thorough explanation about the purpose of the study, which was approved by the local institutional ethics committee.

In the clinical and biochemical workout hyperCKemia was found (1,786 U/l) without any other neurological signs or symptoms. The girl’s pre- and perinatal history of was normal, as was the family history. The parents were not consanguineous. In the recent history of the girl a couple of similar episodes (muscle pain and inability to walk) were reported, both following dancing and skiing without dark urine. Both of the episodes resolved in about 24 h. HyperCKemia was confirmed 1 month later, even without reported exercise in the previous hours (CK 4,446 U/l). Neurological examination was normal. Biochemical study in the family showed high CK in her apparently healthy brother, so a Multiplex Ligation-dependent Probe Amplification (MLPA) analysis of the Dystrophin gene and Dried Blood Spot (DBS) enzyme assay for Pompe disease were carried out, both with negative results. Muscle magnetic resonance imaging was normal: no remarkable signs of muscle disease were found. Spirometry and cardiologic examination did not show any abnormalities. Then a muscle biopsy was performed: it showed slight morphological alterations, possible expression of a previous metabolic rhabdomyolysis, or, alternatively, a paucisymptomatic muscular dystrophy. No increased content of glycogen and lipids was seen; myophosphorylase and myoadenylate deaminase activity, as well as the mitochondrial enzyme activity, were normal. Anti-dystrophin antibodies displayed a normal pattern. Muscle biopsy and clinical features suggested a lipid metabolic disorder, and the dosage of CPTII was recommended: enzyme assessment showed a reduced activity. Then, genetic analysis confirmed the diagnosis: deficiency of CPTII, with a mutation in the heterozygosis of p. S113L (typical locus). Subsequent genetic analysis confirmed that the brother carried the same mutation.

### Nutritional Approach, Metabolic, and Functional Examination

After diagnosis, the use of a weekly food diary template highlighted consumption of fats as well as processed foods. A personalized low-fat normocaloric diet of 2,000 kcal/day was achieved based on the estimated calorie needs per day, by age, gender, and physical activity level (Corkins et al., 2016). A nutrition follow-up was set up for 3 months before the metabolic and functional assessments. The daily diet was tailored to reach a macronutrient intake of around 65% carbohydrates, 20% fat, and 15% protein, as was suggested by a previous study on this topic (Ørngreen et al., 2003). Carbohydrate intake included medium and low glycemic index (GI) foods (e.g., whole grain bread, pasta, oatmeal, selected fruits, etc.), with limitation of high GI foods (e.g., sweets in general), to prevent post-absorptive hyperinsulinemia, which impairs glycogenolysis, which represents a fundamental energy supply process in CPTII-deficient patients (Ørngreen et al., 2003). To support an adequate muscle protein metabolism, the total protein intake, mainly based on high quality protein/low-fat foods (e.g., poultry, low-fat fish, legumes, skim milk, egg whites, etc.), was distributed in each daily meal (breakfast, lunch, dinner, and snacks). Dietary fat intake was based on the use of extra virgin olive oil (one or two servings per day), as a primary fatty acid source, with a restriction of foods rich in saturated fatty acids and cholesterol (e.g., fatty cheese, fast-foods, low nutrition quality snacks, etc.). Furthermore, the use of medium-chain triglycerides oil was also suggested (one serving per day), in relation to its capacity to stimulate mitochondrial biogenesis and metabolism through the activation of Akt and AMPK and inhibition of TGF-β signaling pathways (Ying et al., 2018).

After 3 months of adherence to the new diet regimen, the resting metabolic rate (RMR) was measured using indirect calorimetry technique (Quark PFT, Cosmed, Italy) under optimal environmental conditions (23°C at 8:00 a.m. in fasting conditions). RMR provides information in the form of oxygen consumption (VO_2_), carbon dioxide production (VCO_2_), and respiratory quotient (RQ = VCO_2_/VO_2_ in steady state conditions), considered an index of which type of fuel is mainly metabolized during the test (RQ = 1 cho, RQ = 0.7 fats) (Guthrie et al., 1964). Gas exchange and metabolic variables were measured continuously using the breath-by-breath method. Following an overnight fast, the participant was instructed to lie down quietly for 10 min, after which a ventilated hood was placed over the head (Canopy, Cosmed, Italy). The subject was asked to relax but remain awake for the duration of the test (25 min). The dilution rate was adjusted during the first 5 min of the test so the fraction of expired VCO_2_ was between 1.0 and 1.2%. This portion of the test was excluded to allow breathing and dilution rate to normalize; RMR and RQ were averaged over the remaining 20 min. The inter-assay coefficient of variation (CV) was 6.5%. Initial RMR was 1,597 kcal/day, with an RQ of 1.07 (normal range at rest 0.7–0.85), highlighting a high percentage of carbohydrates utilization (92%).

Further, a cardiopulmonary exercise test (CPET) was performed on a cycloergometer (E 100, Cosmed, Italy), with the patient wearing a two-way breathing mask covering the nose and mouth (V2 Mask TM, Hans Rudolph Inc., United States), connected to a gas analyzer (Quark PFT, Cosmed, Italy). CPET was performed with an incremental technique (10 W every 30 s, with a previous warm up of 3.5 min at 25 W), and VO_2_ and VCO_2_ output were measured using the breath-by-breath method. The test was performed until exhaustion, with the achievement of a respiratory exchange ratio (RER) of 1.16 (maximal effort index). The maximal power reached was 120 watts. VO_2_peak was 1,693 ml/min corresponding to 21.7 ml/min/kg (normal range for age and gender over 1,933 ml/min or over 24.8 ml/min/kg). The heart rate peak (HRpeak) was 153 bpm, 75% of the predicted HRpeak.

Overall CPET underlined a very low oxygen uptake during the effort, not from respiratory deficiency, as VE/VCO_2_ was below 30, but from the metabolic impairment.

### Exercise Intervention

After getting used to the proposed exercises, the patient was involved in an unsupervised 1 h exercise training session three times per week for 6 months. Each session included interval training and resistance exercise activity. The interval training consisted of a 1 min run and 5 min walk for 15 min progressively increased to 30 min (0° inclination). During the patient’s running, the heart rate, monitored with a cardiofrequenzimeter, was maintained over 70% HRmax (corresponding to a RER of 0.98 measured at the first CPET). Resistance training was based on three sets of eight repetitions with 2 min rest between sets. During training, upper/lower split workouts were carried out. Upper-body exercises included bench press, shoulder press, lat pulldown, dumbbell flies, and dumbbell pull-over and arm curls. Load was fixed at 6–7 of the 0–10 scale for resistance exercise (Robertson et al., 2003) according to Herrera-Olivares et al. (2019).

Blood CK was checked before and 36 h after two training sessions chosen randomly with the following results: pre-313 U/L, post-314 U/L; pre-439 U/L, post-242 U/L). Before, during, and after each session, the patient drank a total of 500 ml of a solution containing 22.4 g of carbohydrates, 3 g of creatine, 248 mg of sodium, and 146 mg of potassium (Isonam Energy, NAMEDSPORT, Italy).

## Results

**FIGURE 1:**
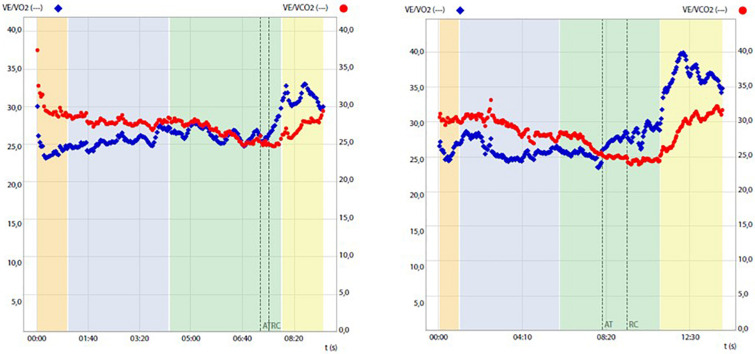
Ventilatory equivalent for oxygen (VE/VO_2_) and for carbon dioxide (VE/VCO_2_) change during cardiopulmonary tests before (left panel) and after (right panel) training, highlighting prolongation of the isocapnic buffering period between the anaerobic threshold (AT) and respiratory compensation point (RCP).

Before and after training RMR and CPET results are reported in Table 1 and Figure 1. After training a slight increase in body weight (+1 kg) and body mass index (BMI) was mirrored by an overall improvement of body metabolism and of the aerobic fitness was found. In particular, RMR increased (+8.1%), indirectly suggesting an increase of metabolically active muscle mass, and RER lowered into the physiological range (from 1.07 to 0.85 at rest; normal range 0.7–0.85). CPET at a maximum (RER achieved 1.18) highlighted an increase of peak power output (+16.7%), aerobic performance (VO_2_peak, 8,3%) and anaerobic threshold (+5.7%), oxygen pulse (+4.5%) and longer isocapnic buffering period (IB) (+335%) (Figure 1). VE/VCO_2_ was still under 30 (25.3) underlining the absence of respiratory deficiency (Table 1).

**TABLE 1:** Summary of anthropometric data and results obtained with indirect calorimetry and cardiopulmonary tests before and after training.

**PRE training (T0)**		
Weight (kg)	78	
Height (cm)	172	
BMI (kg/m^2^)	26.4	
RMR (kcal/day)	1,597	
RQ	1.0	

	**Anaerobic threshold**	**Peak exercise**

Time	7:15	7:55
Workload (W)	100	120
Workload (MET)	4.8	6.2
VO_2_ (ml kg^–1^ min^–1^)	16.6	21.7
RER	1	1.16
HR (bpm)	154	153
VO_2_/HR (ml/beat)	8.4	11.1
IB (sec)	17	

**POST training (T1)**		

Weight (kg)	79	
Height (cm)	172	
BMI (kg/m^2^)	26.7	
RMR (kcal/day)	1,726	
RQ	0.85	

	**Anaerobic threshold**	**Peak exercise**

Time	8:08	10:59
Workload (W)	90	140
Workload (MET)	5	6.6
VO_2_ (ml kg^–1^ min^–1^)	17.4	23.2
RER	1.03	1.18
HR (bpm)	131	159
VO_2_/HR (ml/beat)	10.5	11.5
IB (s)	74	

*BMI, body mass index; RQ, respiratory quotient; RMR, resting metabolic rate; VO*2*, oxygen consumption; RER, respiratory exchange ratio; HR, heart rate; VO_2_/HR, oxygen pulse; IB, isocapnic buffering period.*

## Discussion

This study demonstrated that under the regimen of a properly balanced diet based on a high consumption of carbohydrates and a low intake of fat, a training program with high intensity/low duration bouts of exercise is safe and associated with an overall improvement of body metabolism and aerobic fitness in a CPTII-deficiency patient.

Initial RMR and cardiopulmonary tests revealed high carbohydrate utilization at rest and scarce aerobic fitness, below what was predicted for age and gender. Following exercise training combined with diet adjustment, RMR increased and RQ decreased toward normalization, suggesting an improvement of metabolic active muscles combined with the optimization of macronutrient utilization. In light of the genetic nature of the impaired fatty acids metabolisms, the observed change in RQ probably reflects an increased efficiency in the energy disposal and utilization of carbohydrates at rest and/or an amelioration of the residual fat utilization through alternative pathways (Joshi and Zierz, 2020).

Similarly, what was recently found by Herrera-Olivares et al. in a case of very long-chain acyl-CoA dehydrogenase (VLCAD) deficiency (Herrera-Olivares et al., 2019), following an exercise program chosen to maintain a high demand for glycolysis and therefore to avoid the dependence of lipolysis on energy production, the patient experienced increases in all aspects of CPET, including time to exhaustion, workload, VO_2_peak, VO_2_ at the anaerobic threshold (AT), and oxygen pulse and isocapnic compensation. Given the results obtained, we can speculate that both muscular (including mitochondrial content and capillary density) and cardiovascular adaptations, including changes in cardiac contractility, blood volume, and cardiac output, may have contributed to the observed VO_2_peak improvement. This possibility is mirrored by concomitant changes in resting RQ, which may justify significant amelioration of the mitochondrial expression and/or function and increased oxygen pulse known to represent an indirect indicator of the cardiac stroke volume (Gropper et al., 2012). Importantly, exercise training was also significantly associated with a broadening of the isocapnic buffering period, which ranges between AT and respiratory compensation point (RCP), which is characterized by a progressively increasing production of lactic acid, physiologically buffered by bicarbonates, and strictly relates to the endurance performance of the subject (Tanaka and Seals, 2008).

In our experimental conditions, the temporal elongation of IB may reflect the optimization of exercise-induced buffering capacity probably due to the amelioration of aerobic fitness, i.e., positively correlated IB phase and VO_2_peak (Oshima et al., 1997).

Importantly, our results also indicated that the training protocol was safe, as neither significant post-exercise changes in blood CK nor muscle pain or rhabdomyolysis was experienced by the patient all along the experimental period. The main pathophysiological mechanisms involved in the progression of rhabdomyolysis include an increase of calcium (Ca^2+^) into the muscle cell. Under resting conditions, Ca^2+^ cellular concentration should remain at nanomolar levels. Ca^2+^ would increase through cell activation and muscle contraction when ATP storage is depleted (Lee, 2010). Several conditions are associated with intracellular rise in Ca^2+^ including the depolarization of the sarcolemma and T-tubule due to an action potential followed by calcium release from the sarcoplasmic reticulum (SR), activation of Na^+^-Ca^2+^ membrane exchanger, and rupture of the sarcolemma (Kim et al., 2016). An increase in intracellular Ca^2+^ has been reported to increase the protease and lipase activities that determine sarcolemma damage. In a context in which lipid oxidation is not efficient, as in CPTII, an increased exercise-induced susceptibility to rhabdomyolysis is expected (Roe et al., 2008). In fact, patients with CPTII deficiency experience recurrent episodes of severe muscle pain associated with rhabdomyolysis together with extreme rise of serum CK and myoglobinuria. In this context, exercise and fasting were proven triggering factors, whereas exposure to cold, infections, low fluid intake, psychological stress, and lack of sleep were equally important (Joshi et al., 2019). In our experimental conditions, a physical activity of short duration that mostly relies on the anaerobic systems may provide sufficient ATP to sustain the increased metabolic request, and this may contribute to avoiding the systemic shock often induced or associated with prolonged efforts and endurance activities at low-to-moderate intensities that mostly depend on lipolysis (Berardinelli and D’Antona, 2019).

## Summary and Limitations

The results of this study show that, after diet adjustment, a training protocol based on high intensity/low duration contractions is safe and may improve metabolism and aerobic fitness in patients affected by CPTII deficiency. Nevertheless, as a single case report, the results must be interpreted with caution and cannot be generalized to other patients, and future studies with proper statistical analysis are needed to confirm the findings. Moreover, we considered a young female patient, and we cannot exclude the possibility that different outcomes may be obtained from training male CPTII deficient subjects, who experience more severe and frequent clinical presentation of the disease (Joshi and Zierz, 2020).

## Data Availability Statement

The raw data supporting the conclusions of this article will be made available by the authors, without undue reservation.

## Ethics Statement

The studies involving human participants were reviewed and approved by Institutional Review Board. Written informed consent to participate in this study was provided by the participants’ legal guardian/next of kin.

## Author Contributions

MP analyzed and interpreted the data, wrote the first draft of the manuscript. EP provided medical care, testing, and medical tracking of the patient. LC supervised functional measurements and interpreted the data. SA analyzed and interpreted the data. MN provided nutritional assessment and correction. MB contributed to writing and revision of the manuscript for intellectual content. AB provided medical care, testing, and medical tracking of the patient, writing and revision of the manuscript for intellectual content. GD’A developed the conception of the study, interpreted the data, and wrote the first draft and the final version of the manuscript. All authors contributed to the article and approved the submitted version.

## Conflict of Interest

The authors declare that the research was conducted in the absence of any commercial or financial relationships that could be construed as a potential conflict of interest.
